# Follicular thyroid carcinoma presenting as solitary liver metastasis: a case report

**DOI:** 10.1186/s13256-016-1140-z

**Published:** 2016-12-03

**Authors:** Azhar J. Battoo, Zubaida Rasool, Zahoor A. Sheikh, Altaf G. Haji

**Affiliations:** 1 Surgical Oncology (Head and Neck Services), Sheri Kashmir Institute of Medical Sciences, Srinagar, 190011 India; 2 Department of Pathology, Sheri Kashmir Institute of Medical Sciences, Srinagar, 190011 India; 3 Surgical Oncology, Sheri Kashmir Institute of Medical Sciences, Srinagar, 190011 India

**Keywords:** Thyroid follicular carcinoma, Liver metastasis, Positron emission tomography, TTF1, Case report

## Abstract

**Background:**

Distant metastasis from differentiated thyroid carcinoma at presentation is rare and isolated liver metastasis on presentation is almost unknown. We report a case of primary follicular carcinoma of the thyroid with isolated liver metastasis at presentation.

**Case presentation:**

A 65-year-old man of Kashmiri origin presented to our tertiary referral center with obstructive jaundice; he was evaluated with magnetic resonance cholangiopancreatography and positron emission tomography-computed tomography. Positron emission tomography-computed tomography documented a lesion in his liver in addition to a metabolically active thyroid nodule. Fine needle aspiration cytology of the liver lesion supplemented with immunohistochemical analysis using thyroid transcription factor 1 confirmed the lesion as being an isolated metastasis from the primary thyroid lesion (which on fine needle aspiration cytology showed follicular architecture).

**Conclusions:**

To best of our knowledge, this is first reported case of primary differentiated thyroid carcinoma presenting with isolated liver metastasis manifesting as obstructive jaundice.

## Background

Differentiated thyroid cancer (DTC) rarely presents with distant metastasis at outset. In a recent Surveillance, Epidemiology, and End Results (SEER) database study that covered a period between 1988 and 2009, a total of 1291 patients with metastatic DTC at diagnosis were identified, amounting to a prevalence rate of 2.2% [1]. Gastrointestinal metastases of thyroid cancer are very uncommon and account for 0.5 to 1% of all distant metastases [2]. Within the gastrointestinal system, the liver is the most common site of secondary spread and accounts for approximately 0.5% of all distant metastases originating from DTC [3]. Massive liver involvement is usually a late finding associated with severe symptomatology and rapid progression to death [4]. Liver metastases usually occur in the setting of widespread metastatic disease. Very few cases (four as per our search of medical databases) of DTC with isolated liver metastases have been reported to date [3, 5–7], but to the best of our knowledge none of these cases presented with obstructive jaundice or with isolated hepatic metastasis. Here we report a newly diagnosed case of follicular thyroid carcinoma with solitary liver metastasis causing cholestasis at presentation.

## Case presentation

**Fig. 1:**
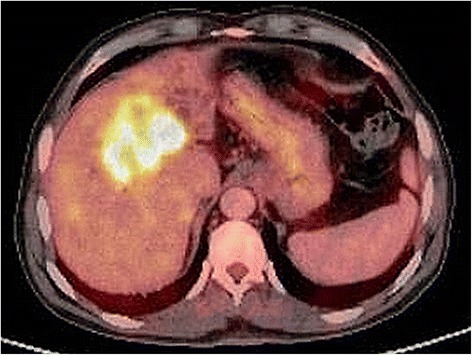
^18^F-fluorodeoxyglucose positron emission tomography-computed tomography image showing a large heterogeneous lesion (measuring approximately 7.2×5.2 cm, maximum standardized uptake value 15.6) in segment IV and adjacent segment II of liver

**Fig. 2:**
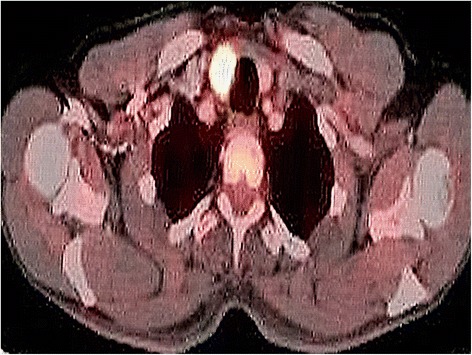
^18^F-fluorodeoxy glucose positron emission tomography-computed tomography image showing hypodense nodule, 1.7×1.7×1.9 cm, with increased uptake (maximum standardized uptake value 32.0) in right lobe of thyroid

**Fig. 3:**
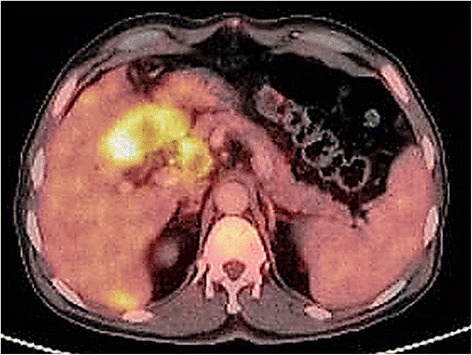
^18^F-fluorodeoxyglucose positron emission tomography-computed tomography image showing 2.9×2.3 cm periportal lymph node with maximum standardized uptake value 7.6

**Fig. 4:**
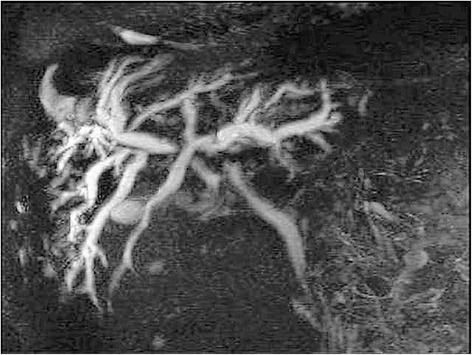
Magnetic resonance cholangiopancreatography showing ill-defined heterogeneous signal intensity mass lesion, predominantly occupying segments II, III, IV, VI, VIII and measuring approximately 7.5×8.5 cm in size

A 65-year-old man of Kashmiri origin with a history of essential hypertension and type II diabetes mellitus was referred to our out-patient department with a diagnosis of obstructive jaundice. He had been already evaluated with a whole body positron emission tomography-computed tomography (PET-CT) scan. The PET-CT scan documented a fluorodeoxyglucose (FDG) avid space-occupying lesion in segments IV and II of his liver that measured approximately 7.2×5.2 cm, maximum standardized uptake value (SUVmax) of 15.6, compressing his left hepatic duct (Fig. 1), along with a metabolically active thyroid nodule (SUVmax 32.0; Fig. 2). In addition, a 2.9×2.3 cm periportal lymph node with SUVmax 7.6 was also noted (Fig. 3). The above findings were confirmed by magnetic resonance cholangiopancreatography (MRCP) which showed an ill-defined heterogeneous signal intensity mass lesion (hyperintense on T2-weighted image and hypointense on T1-weighted image), predominantly occupying segments II, III, IV, VI, and VIII and measuring approximately 7.5×8.5 cm (Fig. 4). The lesion was shown to predominantly involve the left ductal system causing upstream dilatation. Multiple heterogeneous lymph nodes (hyperintense on T2 and diffusion-weighted imaging) were seen in his periportal region. A physical examination of our patient revealed icterus and mild hepatomegaly; palpation of his neck was unremarkable. He had altered liver function tests (LFTs) with grossly elevated bilirubin (total bilirubin 10.1 mg/dl, direct bilirubin 7.2 mg/dl, indirect bilirubin 2.90 mg/dl) and alkaline phosphatase levels. The results of a complete blood count (CBC), coagulation assay, and kidney function tests (KFTs) were normal.

**Fig. 5:**
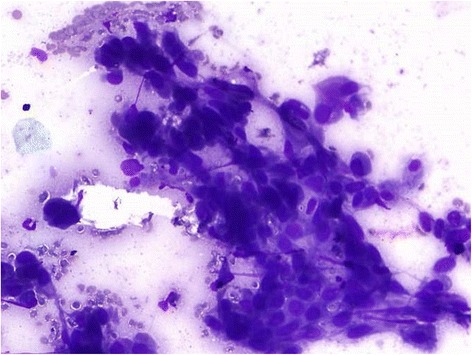
Thyroid aspirate showing many syncytial cell clusters forming follicles, nuclear crowding, and overlapping

**Fig. 6:**
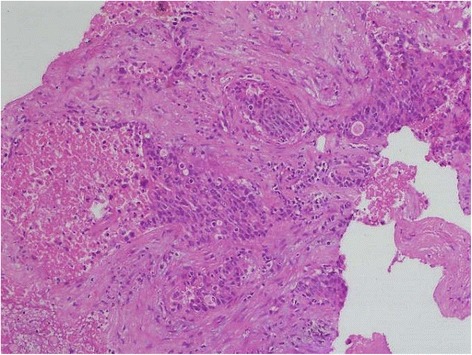
Metastatic deposits of follicular carcinoma in liver with surrounding areas of necrosis. Hematoxylin and eosin, 10×

**Fig. 7:**
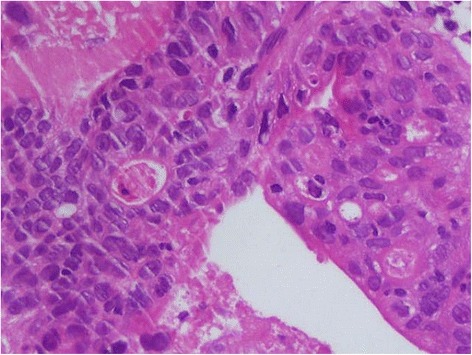
Microfollicular groups, few with abnormal colloid in small central lumen. Cells exhibiting nuclear hyperchromasia and coarse chromatin. Hematoxylin and eosin, 40×

**Fig. 8:**
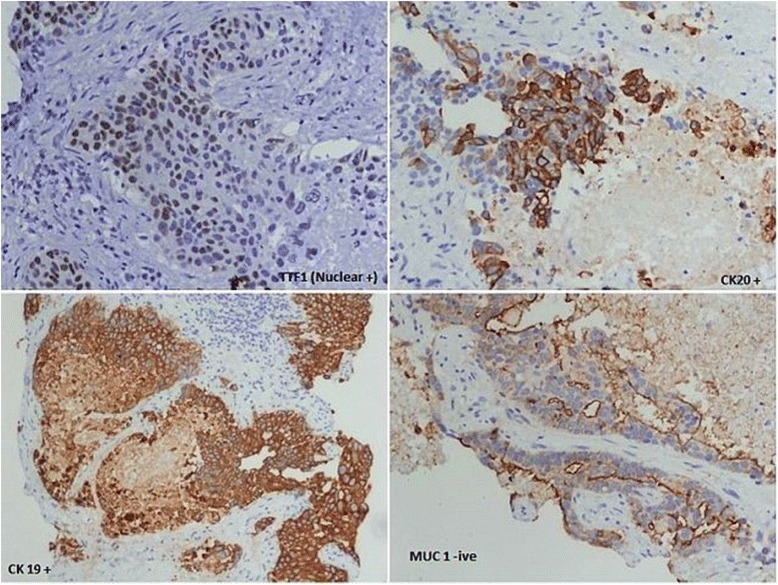
Immunohistochemical staining revealing thyroid transcription factor 1 positive, cytokeratin 19/20 positive, and mucin 1 negative follicular carcinoma. *CK* cytokeratin, *MUC* mucin, *TTF1* thyroid transcription factor 1

Ultrasonography (USG)-guided fine needle aspiration cytology (FNAC) of the liver lesion was reported as papillary adenocarcinoma. FNAC from his thyroid was labelled as follicular neoplasm (Bethesda IV category; Fig. 5). An USG-guided core-cut biopsy of the liver lesion was carried out and hematoxylin and eosin examination of this revealed metastatic deposits of follicular carcinoma with surrounding areas of necrosis (Figs. 6 and 7). Immunohistochemical staining of the liver biopsy was positive for thyroid transcription factor 1 (TTF-1), cytokeratin (CK) 19 and CK20, and negative for mucin (MUC) 1 (Fig. 8), establishing his thyroid as the primary source of the liver lesion.

In view of PET-CT and pathological findings he was finally diagnosed to have follicular carcinoma of the thyroid with isolated hepatic metastasis with obstructive jaundice and was scheduled for total thyroidectomy followed by radioactive I^131^ ablation (RIA). In view of his altered LFTs, it was decided to subject him to preoperative biliary drainage by biliary stenting. However, unfortunately, post-procedure he developed acute cholangitis and severe sepsis which proved fatal for him.

## Discussion

Isolated liver metastasis from DTC is rare and very few cases have been reported in the medical literature. Our search of medical databases revealed that only four cases of DTC with isolated liver metastases have been reported in the English medical literature until now [3, 5–7]. It is to be noted that none of the cases reported had liver metastasis at the time of presentation; hence, ours is the first reported case of DTC with isolated liver metastasis at presentation which was complicated with obstructive jaundice.

Graves *et al.* [5] reported a case of 36-year-old man with an incidental finding of painless left thyroid nodule who subsequently underwent a subtotal thyroidectomy. A pathologic review of the excised tissue revealed a 3‐cm follicular carcinoma of his left thyroid lobe with capsular and vascular invasion. Immunohistochemical staining was positive for thyroglobulin (Tg) and negative for calcitonin. His serum Tg level was 25,000 ng/ml. A postoperative I^131^ scan (5 mCi) demonstrated uptake in his thyroid bed with no evidence of distant metastases. There were no foci of abnormal activity to suggest distant metastases. He was treated with 100 mCi I^131^ for ablation of the remnant thyroid tissue. A follow‐up examination 9 months after the initial treatment revealed a Tg level of 17,000 ng/ml. A bone scan and chest X‐ray demonstrated normal findings with no evidence of bone or lung metastases. A follow‐up I^131^ examination (5 mCi I^131^) showed an intense focal area of abnormal activity in the region of his liver, suspicious for liver metastasis.

Kondo *et al*. [6] reported a case of 48-year-old Japanese woman who was operated (subtotal thyroidectomy) for follicular adenoma of her thyroid. Eight years after the initial surgery, an isolated liver lesion was incidentally detected on abdominal computed tomography (CT) scan. The liver lesion was resected with a cuff of liver parenchyma and subjected to histopathological evaluation. The tumor lacked a capsule and had an ill-defined border with the liver tissue. On histological examination, the nodule was entirely composed of small to large follicles containing colloid material. These follicles were lined by flattened-to-cuboidal cells, having uniform and round nuclei and non-remarkable nuclear atypia. Immunohistological studies confirmed Tg, triiodothyronine (T_3_), and thyroxine (T_4_) positivity, consistent with follicular cells or colloid material. No mitotic activity and a low MIB-1 positive index (<1% positive cells) were noted in the specimen. Staining of p53 was negative. Histopathology of her thyroid specimen (subtotal thyroidectomy done 8 years earlier) was reviewed and additional sections revealed a focus of tumor thrombus in capsular vessel which was completely enveloped by the vessel wall endothelium. Thus she was diagnosed to have had minimally invasive follicular carcinoma with secondary isolated liver metastasis.

Salvatori *et al*. [3] described a similar case with a solitary liver metastasis from Hürthle cell thyroid cancer, which appeared during long-term follow-up. The lesion was diagnosed by progressive increase of serum Tg and confirmed with a positive I^131^ scan, USG of the abdomen, magnetic resonance imaging (MRI) of the liver, and ^18^F-FDG-PET scan.

Kouso *et al*. [7] reported follicular thyroid carcinoma in a 73-year-old woman who had undergone curative resection of thyroid carcinoma 32 years earlier. CT of her abdomen revealed a round lesion, approximately 1.5 cm in diameter, which was enhanced early and washed out later, in segment 5 of her liver. She underwent laparotomy and partial resection of her liver. Microscopic examination showed follicular cells with minimal atypia growing in a thyroid follicular pattern with colloid, whereby a diagnosis of metastatic liver cancer from thyroid follicular carcinoma was made.

The discussion of the above four cases reveals that all of them were recurrent cases of thyroid carcinoma. Ours is the first reported case of primary thyroid carcinoma presenting with isolated liver metastasis and complicated by obstructive jaundice.

For patients with a Tg level above some arbitrary limit, the administration of a large dose (3.7 to 5.5 GBq; 100 to 150 mCi) of I^131^, in order to obtain a highly sensitive therapeutic whole body scan (WBS), remains the best diagnostic strategy. However, on very rare occasions, physiological enteric radioactivity can hide possible abdominal lesions and further in-depth studies, such as FDG-PET scans, are sometimes necessary.

Although immunohistochemical analysis was carried out on the metastatic lesion in the case reported by Kondo *et al*. [6], none of the other three reported case had IHC done on the metastatic lesions. In our study, IHC was done with TTF-1 marker, which was reported as strongly positive. TTF-1 being specific to only thyroid and lung tissue and with no lung lesion visualized on imaging studies, diagnosis of follicular thyroid carcinoma with isolated liver metastasis at presentation was made.

## Conclusions

The present case exemplifies a very rare presentation of DTC; however, advanced imaging techniques combined with appropriate immunohistochemistry should be used before labelling liver lesion as metastatic from a rare primary site, as was done in our case.

## Abbreviations

CT: Computed tomography
DTC: Differentiated thyroid cancer
FDG: Fluorodeoxyglucose
FNAC: Fine needle aspiration cytology
IHC: Immunohistochemistry
LFTs: Liver function tests
PET/CT: Positron emission tomography-computed tomography
SUVmax: Maximum standardized uptake value
Tg: Thyroglobulin
TTF-1: Thyroid transcription factor 1
USG: Ultrasonography.
